# A Peptide Amphiphile Organogelator of Polar Organic Solvents

**DOI:** 10.1038/srep43668

**Published:** 2017-03-03

**Authors:** Charlotte K. Rouse, Adam D. Martin, Christopher J. Easton, Pall Thordarson

**Affiliations:** 1 Research School of Chemistry, The Australian National University, Canberra, ACT 2601, Australia; 2 School of Chemistry, The Australian Centre for Nanomedicine and the ARC Centre of Excellence in Convergent Bio-Nano Science and Technology, The University of New South Wales, Sydney, NSW 2052, Australia

## Abstract

A peptide amphiphile is reported, that gelates a range of polar organic solvents including acetonitrile/water, *N*,*N*-dimethylformamide and acetone, in a process dictated by β-sheet interactions and facilitated by the presence of an alkyl chain. Similarities with previously reported peptide amphiphile hydrogelators indicate analogous underlying mechanisms of gelation and structure-property relationships, suggesting that peptide amphiphile organogel design may be predictably based on hydrogel precedents.

Peptide-based supramolecular gels are an important class of self-assembling materials that can be defined as organogels^1^,^2^,^3^ or hydrogels^4^,^5^,^6^, based upon the type of solvent encapsulated. They are attractive candidates for biomedical applications such as for use in three-dimensional cell-culture scaffolds^7^,^8^,^9^,^10^, as they retain a high solvent content whilst being able to mimic the fibrous nature of the extracellular matrix. The use of peptides as gelators confers even greater levels of biocompatibility as bioactive sequences can be introduced^11^,^12^,^13^, and they are generally non-toxic. Whilst most of the emphasis has been on hydrogels, cheap, easily synthesized and non-toxic organogels have also found important applications in areas such as drug delivery, the immobilization of hazardous liquids for handling and transport, and the containment of chemical spills^14^,^15^,^16^,^17^,^18^. Organogels which gel in the presence of polar organic solvents such as acetonitrile, acetone and *N,N*-dimethylformamide have been used in a variety of applications, from matrices for crystallization of active pharmaceutical ingredients^19^,^20^, to colorimetric anion sensors^21^ to light-responsive gels^22^.

As part of a conformational study of polyalanines and polyvalines, the peptide sequence VVVGHVVV was found to be peculiarly soluble in water and, surprisingly, to form a β-sheet assembly in water-acetonitrile mixtures but not in water alone. As it was noted that the peptide VVVGHVVV has a similar sequence pattern to previously reported peptide amphiphile (PA) hydrogels, the alkyl-chain modified peptide VVVGHVVV-C_8_ was prepared, and the gelation behavior of the peptide and its alkyl derivative was investigated. PAs featuring azobenzene and adamantane at the *N*-terminus of the VVVGHVVV peptide were also synthesized, however only the alkyl-modified peptide exhibited gelation behavior. Hydrogel-forming PAs, such as EEEAAAVVV-C_16_ (Fig. 1) which has been studied by the Stupp^23^,^24^,^25^,^26^ and Nguyen^27^,^28^ groups, consist of an alkyl chain, a peptidic β-sheet assembling region and a hydrophilic head group. These PAs are understood to aggregate *via* association of their hydrophobic tails to form a micellar core which stacks along the fiber axis, stabilized by β-sheet interactions which continue along the length of the fiber. The charged head group affects water solubility and gelation^29^,^30^.

The gelation behavior of VVVGHVVV-C_8_, and the structure and mechanical properties of its gels, were investigated using atomic force microscopy (AFM), rheology, circular dichroism (CD) spectroscopy, and Fourier transform infrared (FT-IR) spectroscopy. We now report that unlike other PAs, VVVGHVVV-C_8_ is not soluble in water and is therefore unable to form a hydrogel, but does form organogels in polar organic solvents such as *N,N-*dimethylformamide (DMF) and acetone, and we analyze the basis of this gel formation.

## Results and Discussion

### Synthesis and Gel Characterization

VVVGHVVV-C_8_ was obtained using solid-phase peptide synthesis, using octanoic acid to acylate the *N*-terminus. For comparison, VVVGHVVV without the *N-*alkyl chain, was also examined. VVVGHVVV-C_8_ displays very limited solubility in water regardless of pH, temperature and sonication, and is therefore unsuitable to form a hydrogel. VVVGHVVV-C_8_ is also insoluble in acetonitrile, however with sonication it dissolves in mixtures of acetonitrile and water, which were therefore used to study gelation. The minimum gel concentration (MGC) in a mixture of 50% acetonitrile/water (v/v) was determined to be 0.06 wt.% (0.64 mM). Thereafter, 0.20 wt.% (2.14 mM) solutions of VVVGHVVV-C_8_ in 10, 20, 30, 40 and 50% acetonitrile/water were used to study gelation, at room temperature. By the inversion test, gelation occurred after 10, 8, 8 and 5 days, and in less than 1 day, for these solutions respectively, showing that more acetonitrile resulted in faster gelation. Increasing the concentration of VVVGHVVV-C_8_ also sped up gelation with 0.8 wt.% (8.56 mM) solutions in 50:50 acetonitrile/water forming gels almost immediately after initial sonication. Since the 0.20 wt.% 50% solution formed most readily, it was used as the focus for further studies. The unalkylated VVVGHVVV was also soluble in acetonitrile/water mixtures, however these solutions did not gelate.

**Figure 2:**
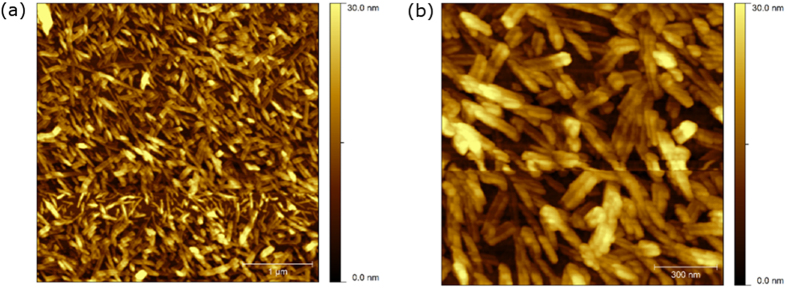
AFM images of VVVGHVVV-C_8_ xerogel from 0.20 wt.% (2.14 mM) solutions of 50% acetonitrile/water at (a) 1 μm (left) and (b) 300 nm (right) scale.

**Figure 3:**
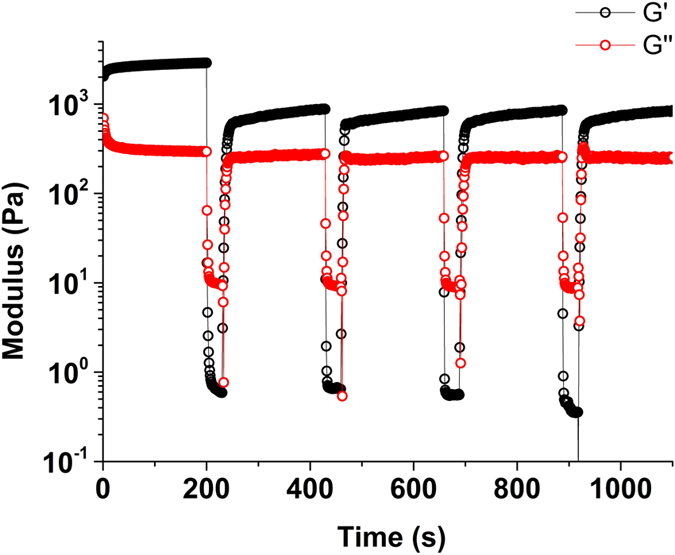
Thixotropic test of the 0.20 wt.% (2.14 mM) gel of VVVGHVVV-C_8_ in 50% acetonitrile/water.

AFM of the gel of VVVGHVVV-C_8_ (Fig. 2a) shows short fibrils ranging in height from 8–12 nm. These are characterized by a valley along the length of each fibril (Fig. 2b), suggesting collapse of the core probably as a consequence of solvent loss during sample preparation. Strain sweep rheological experiments performed on the gel (Fig. S8) formed from a 0.20 wt.% solution at a constant frequency of 1 Hz, showed that the mechanical properties deviated from the linear viscoelastic region at less than 1% strain, and the cross-over point from gel to liquid (where the storage modulus G′ = the loss modulus G″) occurred at 3% strain. An oscillatory frequency sweep performed (Fig. S9) at a constant 0.2% strain demonstrated that the gel is frequency independent over the range studied, with a stiffness (approximated by G′) of 1.5 × 10^5^ Pa. VVVGHVVV-C_8_ therefore forms a very stiff but brittle gel. A thixotropic test, with application of a low strain (0.2%, 200 seconds) followed by a large strain (100%, 30 seconds), acts to completely liquefy the gel network, and upon repetition of this strain sequence, shows that the recovery of the mechanical properties of the gel is fast but incomplete (Fig. 3).

**Figure 4:**
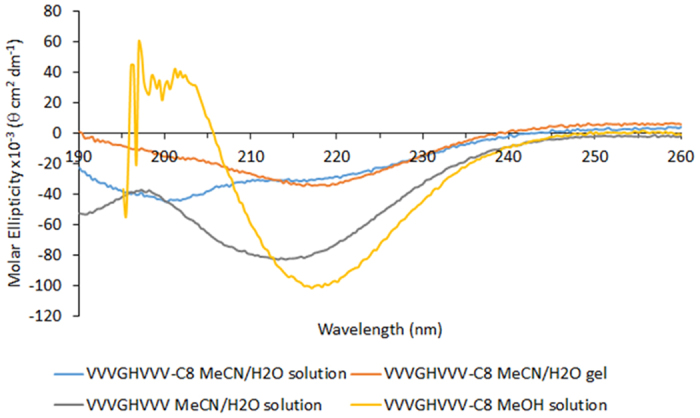
Circular dichroism spectra of VVVGHVVV-C_8_ 50% acetonitrile/water solution, VVVGHVVV-C_8_ 50% acetonitrile/water gel, VVVGHVVV in 50% acetonitrile/water solution and VVVGHVVV-C_8_ in methanol solution.

The CD spectrum of the gel (Fig. 4) shows a minimum at 218.3 nm, characteristic of β-sheet secondary structure^31^. The spectrum of a corresponding solution of VVVGHVVV-C_8_ before gelation occurs shows a minimum at 200.9 nm with a shoulder at ∼215 nm, indicating a mixture of random coil/disordered and β-sheet structures, respectively. The differences between these spectra show that gelation involves rearrangement of these disordered structures into a β-sheet assembly. Even though the unalkylated VVVGHVVV does not form a gel, its CD spectrum also shows an intense minimum at 214.1 nm characteristic of β-sheet formation, demonstrating that gelation is not solely determined by interactions between peptide chains and the alkyl moiety of VVVGHVVV-C_8_ is key to gel formation.

The structural information provided by CD spectroscopy is mirrored in results obtained using FT-IR spectroscopy. The gel of VVVGHVVV-C_8_, prepared in 50% acetonitrile/D_2_O instead of acetonitrile/water so as to eliminate interference from H_2_O^32^, showed an amide I carbonyl signal at 1624 cm^−1^ corresponding to a β-sheet structure^33^. The signal at 1626 cm^−1^ in the corresponding spectrum of the unalkylated VVVGHVVV shows a similar structure, again demonstrating that gelation is not only dependent on peptide secondary structure, and that the alkyl moiety of VVVGHVVV-C_8_ is required. The carbonyl signals in the spectra of both the gel of VVVGHVVV-C_8_ and the solution of VVVGHVVV are each red-shifted by approximately 5 cm^−1^, relative to those of the corresponding powders, which appear at 1630 and 1631 cm^−1^, respectively. This provides tentative evidence that the interactions between the peptide chains in the gel of VVVGHVVV-C_8_ are similar to those in the solution of VVVGHVVV.

**Figure 5:**
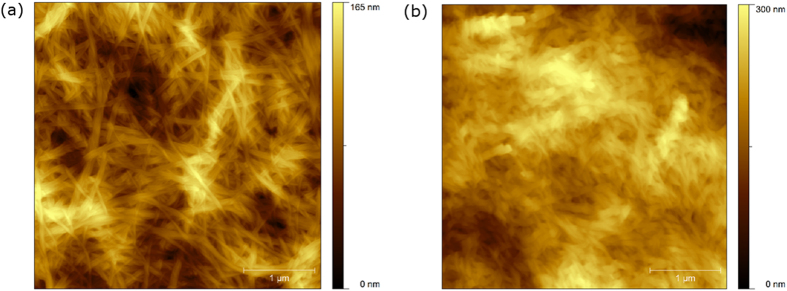
AFM images of VVVGHVVV-C_8_ xerogel from 0.20 wt.% (2.14 mM) solutions in (a) DMF (*left*) and (b) acetone (*right*). Scale bars represent 1 μm.

Considering solvents other than acetonitrile/water, VVVGHVVV-C_8_ did not dissolve in ethyl acetate, diethyl ether or hexane despite heating to reflux temperatures and sonicating for up to one hour. VVVGHVVV-C_8_ dissolved in dimethyl sulfoxide, methanol, ethanol, isopropanol, chloroform, dichloromethane and toluene but did not form gels from those solutions, and dissolved in DMF and acetone to give solutions that form gels with MGCs of 0.06 wt.% in each case. No gelation of VVVGHVVV-C_8_ was observed in methanol even though the CD spectrum shows that strong β-sheets are formed (Fig. 4). AFM of the DMF gel (Fig. 5a) shows fibers that are much longer (several microns) and more bundled (with a larger diameter of 10–20 nm) than the fibrils of the acetonitrile/water gel. In the case of acetone (Fig. 5b), strong fiber bundling is observed, precluding measurements of the fiber diameter.

Circular dichroism measurements of the acetone and DMF organogels were not possible, because of strong solvent absorption obscuring any secondary structure information. FT-IR spectroscopy of the DMF gel was not possible as the carbonyl signal is masked, but FT-IR of the acetone organogel shows a carbonyl stretch at ∼1629 cm^−1^ (Fig. S10 and S11), representative of a β-sheet structure. This suggests that the secondary structure of VVVGHVVV-C_8_ is preserved when the gelating solvent is changed. Rheology experiments (Figs S14 and S15) on the VVVGHVVV-C_8_ DMF organogel show that it is two orders of magnitude weaker than the corresponding water/acetonitrile gel, but more amenable towards oscillatory strain, with no cross-over point observed. No previously reported DMF organogels^34^,^35^,^36^ approach the gel stiffness (taking into account relative wt.%) or minimum gel concentrations observed in this study, making it unique.

### Basis of Gel Formation

As previously discussed, VVVGHVVV-C_8_ is structurally similar to PAs that form hydrogels, in that it comprises an alkyl chain, a peptide region and a charged head group. Even so, it is unsuitable for hydrogel formation because it does not dissolve in water, presumably because its head group is less polar than, for example, that of EEEAAAVVV-C_16_. It is however, soluble in organic solvents and that provided the opportunity to explore its organogel behavior and observe acetone, DMF and acetonitrile/water gels. Organogelators that comprise a peptide component are already known but they do not have the other components of PAs^37^,^38^,^39^. Some do form β-sheets^35^,^40^,^41^, but gelation generally depends on π-π-interactions^36^,^42^,^43^.

CD and FT-IR spectra show that gelation of VVVGHVVV-C_8_ involves β-sheet formation. Since VVVGHVVV also forms β-sheets without forming gels however, it is clear that these interactions are not the sole basis for extended fiber formation, and both the alkyl chain and peptide component are required. With hydrogelating PAs it has also been observed that an alkyl chain of more than six carbons is necessary for gelation to occur^44^. Although the details of the gelation of VVVGHVVV-C_8_ are not delineated by our studies, the analogy with PAs such as EEEAAAVVV-C_16_ suggests a similar gelation mechanism despite the difference in solvent; that is micelle-type hydrophobic aggregation of alkyl chains which propagates down the fiber axis, then reinforced by peptide β-sheet interactions extending along the length of the fiber.

The stiffness of the acetonitrile/water gel of VVVGHVVV-C_8_ (1.5 × 10^5^ Pa) is higher than that of similar PAs such as EEEAAAVVV-C_16_ (<10^4^ Pa). This is consistent with previous findings that increasing the proportion of valines in the β-sheet forming region of a PA results in an increase in gel stiffness^26^. With PAs such as EEEAAAVVV-C_16_, the β-sheet signal in the CD spectrum is red-shifted by a magnitude that inversely correlates with gel stiffness, and this has been attributed to the extent of twisting of the sheets, i.e., stiffer gels involve stronger peptide interactions with the least twisting^26^. Again, the gel of VVVGHVVV-C_8_ fits this trend. The corresponding signal is red-shifted by only 2.3 nm, consistent with a very stiff gel.

## Conclusion

In summary, VVVGHVVV-C_8_ is a peptide amphiphile that gelates non-aqueous solvents. Assembly occurs in acetonitrile/water, DMF and acetone *via* β-sheet interactions, facilitated by the presence of the alkyl chain. Analogies with previously reported PA hydrogelators suggest that the underlying mechanism of gelation is the same despite the difference in solvent, and that structure-property relationships identified with these PAs also hold true. This study therefore indicates that the existing extensive literature that reports on PA hydrogels may be drawn upon to usefully inform PA organogel retro-design and predictability, for assembly in a range of solvents.

## Methods

### Peptide Synthesis

VVVGHVVV-C_8_ was obtained using standard solid-phase synthesis with Wang resin preloaded with the first Fmoc-protected amino acid. Resin (0.69 mmol g^−1^, 0.25 mmol, 362 mg) was swollen in DMF (2 mL) for 2 h before being loaded onto a CEM Liberty Automated Peptide Synthesizer. Amino acid solutions in DMF (0.2 M, 4 eq) were used, along with HBTU in DMF (0.45 M, 4 eq) as the activator, DIPEA in NMP (2 M, 3.6 eq) as the activator/base and HOBt in 20% piperidine in DMF (0.1 M, 4 eq) for deprotection. Octanoic acid (0.2 M, 4 eq) was used in the final coupling step, and the final deprotection step omitted. Coupling was performed in the CEM Discover Microwave Unit, with single couplings performed at 70 °C apart from Fmoc-*N*-His(Trt) which was coupled at 50 °C, and octanoic acid which underwent double-coupling. The peptide was then cleaved from the resin with a solution of TIPS (50 μL), water (50 μL) and TFA (4 mL), in which the resin was left for 4 h. The reaction mixture containing the cleaved peptide was filtered, and the filtrate was pipetted into ice-cold, stirring 10% acetonitrile/water (100 mL), to give a precipitate of the crude product that was recovered by centrifugation. VVVGHVVV-C_8_ was subjected to reverse-phase HPLC with a gradient of 50–90% acetonitrile/water over 30 min. Fractions eluting at 5.8 min were combined and lyophilised to yield VVVGHVVV-C_8_ as a colorless solid (52 mg, 22%).

VVVGHVVV-C_8_: *N*-Octanoic acid-Val_3_-His-Gly-Val_3_: m.p. = 220–223 °C dec.; ^1^H NMR (400 MHz, [D_6_]DMSO): δ = 8.70 (s, 1 H), 8.23–7.79 (m, 8 H), 7.28 (s, 1 H), 4.57–4.52 (br s, 1 H), 4.31–4.09 (m, 6 H), 3.85–3.69 (m, 2 H), 3.09–2.93 (m, 2 H), 2.18–1.88 (m, 8 H), 1.49–1.46 (m, 2 H), 1.23 (br s, 9 H), 0.88–0.79 ppm (m, 38 H); ^13^C NMR (100 MHz, [D_6_]DMSO): δ = 172.79, 172.21, 171.19, 171.07, 170.97, 170.93, 170.65, 169.94, 168.51, 129.02, 128.33, 126.23, 57.80, 57.70, 57.63, 57.59, 57.40, 57.02, 51.50, 41.91, 35.12, 31.18, 30.80, 30.27, 30.23, 30.21, 29.73, 28.52, 28.42, 25.41, 22.02, 19.21, 19.11, 19.08, 19.06, 19.04, 18.40, 18.28, 18.19, 18.14, 18.00, 17.87, 13.93 ppm; analytical HPLC (YMC ODS-AQ 150 × 4.6 mm, 3 μm column): *t*_R_ 35.87 min (gradient; MeCN (0.1% TFA): H_2_O (0.1% TFA), 5:95 to 95:5 over 45 min, 0.5 mL min^−1^); HRMS-ESI *m/z* [M + H]^+^ calcd for C_46_H_81_N_10_O_10_, 933.6137; found 933.6141, [M + Na]^+^ calcd for C_46_H_80_N_10_O_10_Na, 955.5957; found 955.5961.

VVVGHVVV was synthesized in the same manner, and the reaction mixture obtained after cleavage from the resin pipetted into ice-cold, stirring diethyl ether (100 mL). The crude product was recovered as a precipitate by centrifugation and subjected to reverse-phase HPLC with a gradient of 5–50% acetonitrile/water over 25 min. Fractions eluting at 16.2 min were combined and lyophilized to yield VVVGHVVV as a colorless solid (38 mg, 19%).

VVVGHVVV*: N-* Val_3_-His-Gly-Val_3_: m.p. = 228–230 °C dec.; ^1^H NMR (400 MHz, D_2_O): δ = 8.67 (s, 1 H), 7.30 (s, 1 H), 4.46 (t, *J* = 6 Hz, 1 H), 4.37–4.05 (m, 6 H), 3.82 (d, *J* = 12 Hz, 1 H), 3.71 (d, *J* = 4 Hz, 1 H), 3.21 (m, 2 H), 2.12–1.84 (m, 6 H), 0.94–0.79 ppm (m, 36 H); ^13^C NMR (100 MHz, [D_6_]DMSO): δ = 172.74, 171.10, 170.90, 170.68, 170.65, 169.95, 168.60, 167.66, 133.74, 129.14, 116.95, 58.03, 57.86, 57.64, 57.53, 57.11, 57.01, 51.39, 41.87, 30.76, 30.42, 30.38, 30.21, 30.03, 29.71, 27.13, 19.14, 19.11, 19.04, 19.01, 18.47, 18.32, 18.17, 18.12, 17.98, 17.92, 17.58 ppm; analytical HPLC (YMC ODS-AQ 150 × 4.6 mm, 3 μm column): *t*_R_ 14.97 min (gradient; MeCN (0.1% TFA): H_2_O (0.1% TFA), 5:95 to 50:50 over 25 mins, 0.5 mL min^−1^); HRMS-ESI *m/z* [M+H]^+^ calcd for C_38_H_67_N_10_O_9_, 807.5092; found 807.5092.

### Gel Formation

VVVGHVVV-C_8_ (0.6 mg) was dissolved in the relevant solvent (1 mL) with sonication (∼60 sec) in a 1.5 mL sealed vial and the mixture was then allowed to stand under ambient conditions. An inversion test established the formation of these 0.06 wt.% gels (0.64 mM). Similarly, VVVGHVVV-C_8_ (1.0 mg) was dissolved in the relevant solvent (0.5 mL) with sonication (∼60 sec) and left under the same conditions to give 0.20 wt.% gels (2.14 mM).

### AFM Imaging

Peptide solutions (0.20 wt.%, 2.14 mM) were made by dissolving in the relevant solution with sonication. Gels were allowed to form, then samples for AFM were prepared by spread coating onto a freshly cleaved mica disc and leaving to dry overnight in ambient conditions to give the xerogel. Imaging was undertaken using a Bruker Multimode 8 in ScanAsyst mode under ambient conditions and with ScanAsyst Air tips (spring constant 0.4 N/m^2^).

### Rheological Measurements

Peptide solutions (0.20 wt.%, 2.14 mM) were made by dissolving in 50% acetonitrile/water with sonication. An aliquot (550 μL) of this solution was then cast onto an Anton Paar MCR 302 rheometer (parallel plate geometry, 25 mm diameter, 1 mm gap) and left to gel over the course of 4 h. A solvent trap and Peltier hood was used to prevent any evaporation during gelation. Frequency sweeps were performed from 0.1–10 Hz, at a constant strain of 0.2% and three repeats performed on separate samples. Strain sweeps were performed from 0.1–100% at a constant frequency of 1 Hz.

### Circular Dichroism Spectroscopy

Solutions of VVVGHVVV-C_8_ (0.20 wt.%, 2.14 mM) were made by dissolving in 50% acetonitrile/water with sonication. These were then diluted by serial dilution with 50% acetonitrile/water until HT < 1,000 across the sample spectra range of 190–260 nm. Spectrometry was performed using a Chirascan CD spectrometer and analyzed using Pro-data software. Samples were measured in a 10 mm Hellma QS cuvette using a 0.3 nm step and 1.5 s per point at 20 °C. The baseline in each case was a blank solution of 50% acetonitrile/water. The average spectra of 5x scans were taken at 27.5 μM, 20.6 μM, 15.5 μM and 11.6 μM and their molar ellipticities then averaged to give the CD spectrum of VVVGHVVV-C_8_ before gelation.

The 2.14 mM solution of VVVGHVVV-C_8_ was left to gel overnight then shaken to give a solution that was diluted by serial dilution with 50% acetonitrile/water. The average spectra of 5x scans were taken at 27.5 μM, 20.6 μM, 15.5 μM and 11.6 μM and their molar ellipticities then averaged to give the CD spectrum of VVVGHVVV-C_8_ after gelation.

A concentrated solution of VVVGHVVV was made in 50% acetonitrile/water and diluted by serial dilutions with 50% acetonitrile/water. The average spectra of 5x scans were taken at 29.06 μM, 21.80 μM, 16.35 μM and 12.26 μM and their molar ellipticities averaged to give the CD spectrum of VVVGHVVV.

A 2.14 mM solution of VVVGHVVV-C_8_ in methanol was made, then diluted by serial dilutions. Spectra in this solvent were measured across a range of 195–260 nm because of the high absorbance of methanol at lower wavelengths. The average spectra of 5x scans were taken at 31.25 μM, 15.6 μM and 7.8 μM and their molar ellipticities then averaged to the CD spectrum of VVVGHVVV-C_8_ in methanol.

### Fourier Transform Infrared Spectroscopy

FT-IR spectroscopy measurements were made on a Perkin Elmer Spotlight 400 FT-IR spectrophotometer equipped with a diamond crystal attenuated total reflectance (ATR) accessory. Solutions and gels were both prepared at 0.20 wt.% (2.14 mM) and pressed between the diamond crystal and substrate. All spectra were scanned 16 times over the range of 4000–650 cm^−1^.

## Additional Information

**How to cite this article**: Rouse, C. K. *et al*. A Peptide Amphiphile Organogelator of Polar Organic Solvents. *Sci. Rep.* **7**, 43668; doi: 10.1038/srep43668 (2017).

**Publisher's note:** Springer Nature remains neutral with regard to jurisdictional claims in published maps and institutional affiliations.

## Supplementary Material

Supporting Information

## Figures and Tables

**Figure 1:**
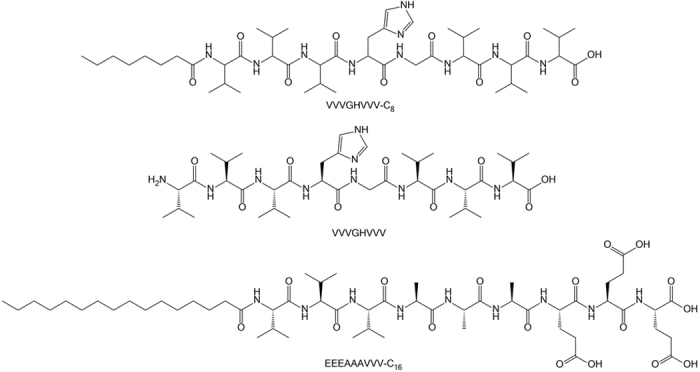
VVVGHVVV-C_8_, the analogous VVVGHVVV without the alkyl chain and EEEAAAVVV-C_16_ which is an example of a hydrogelating species previously reported^18^,^19^,^20^,^21^,^22^,^23^.
