# Supplementary material for: A Peptide Amphiphile Organogelator of Polar Organic Solvents
Source: Sci Rep. 2017 Mar 3;7:43668. doi: 10.1038/srep43668 (PMC5334642; doi:10.1038/srep43668)
Supplement: Supporting Information [file srep43668-s1.pdf]

Supporting Information

A Peptide Amphiphile Organogelator of Polar Organic Solvents

Charlotte K. Rouse,[a] Adam D. Martin,*[b] Christopher J. Easton[a] and Pall Thordarson[b]

[a]Research School of Chemistry, The Australian National University, Canberra, ACT 2601, Australia

[b]School of Chemistry, The University of New South Wales, Sydney, NSW 2052, Australia

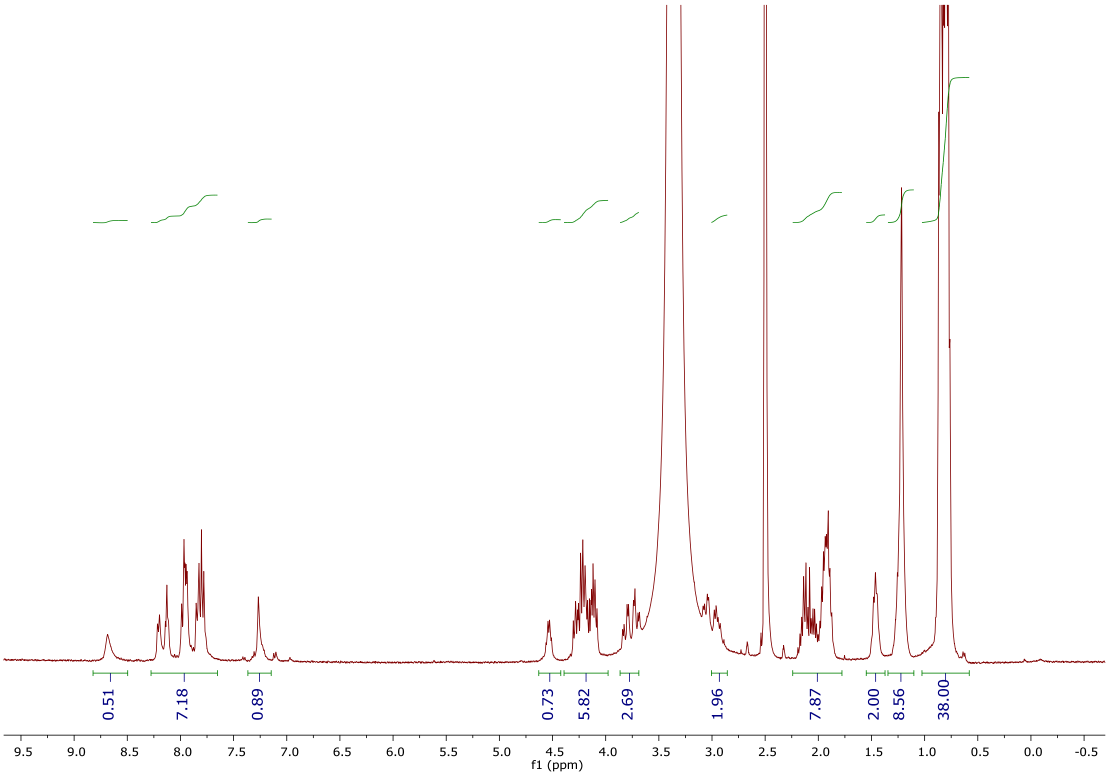

S 1 ^{1}H NMR spectrum of VVVGHVVV-C_8 in D_6-DMSO at 400 MHz

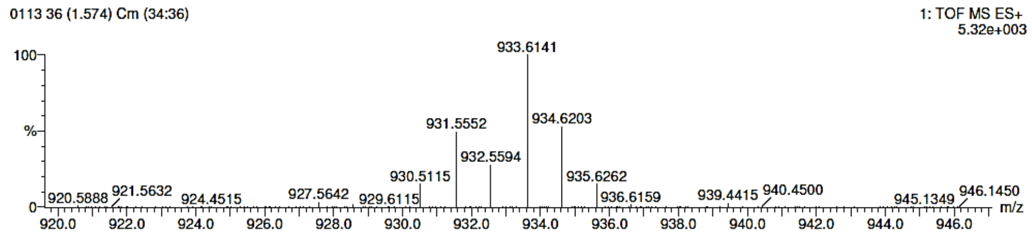

	Mass	Calc. Mass	mDa	PPM	DBE	i-FIT	Formula
Minimum:					-1.5		
Maximum:			5.0	3.0	20.0		
	933.6141	933.6137	0.4	0.4	11.5	12.9	C46 H81 N10 O10

S 2 ESI HRMS of VVVGHVVV-C_8 ion $[M+H]^+$

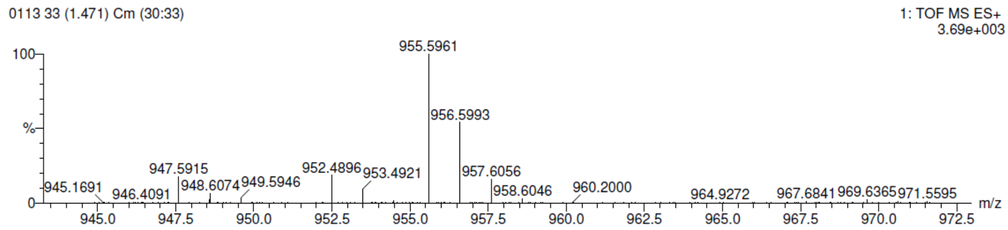

	Mass	Calc. Mass	mDa	PPM	DBE	i-FIT	Formula
Minimum:					-1.5		
Maximum:			5.0	3.0	20.0		
	955.5961	955.5957	0.4	0.4	11.5	3.6	C46 H80 N10 O10 23Na

S 3 ESI HRMS of VVVGHVVV-C_8 ion $[M+Na]^+$

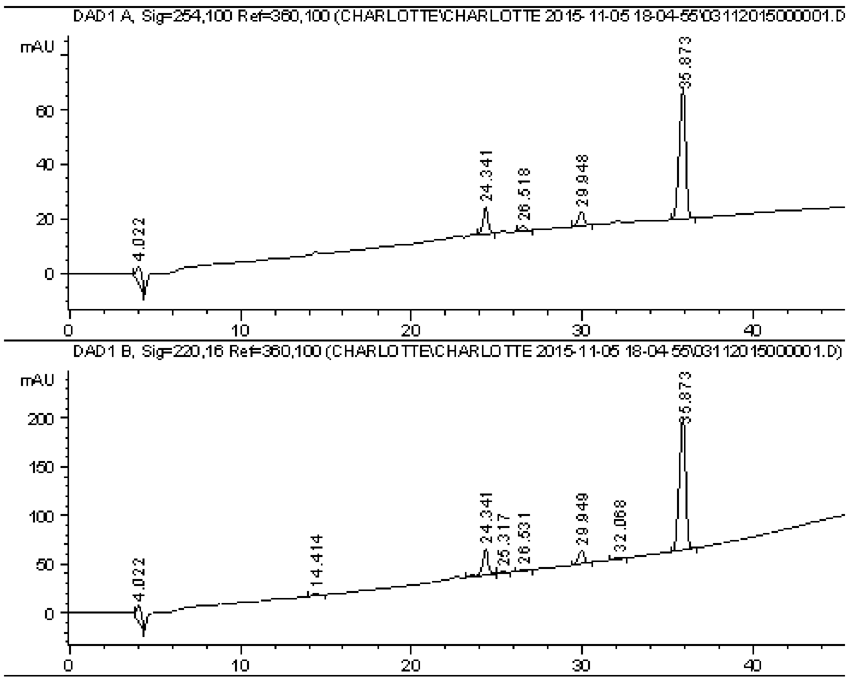

S 4 Analytical HPLC trace of VVVGHVVV-C_8 at 254 and 220 nm. Gradient; MeCN (0.1% TFA): H_2O (0.1% TFA), 5:95 to 95:5 over 45 min, 0.5 mL min^{-1})

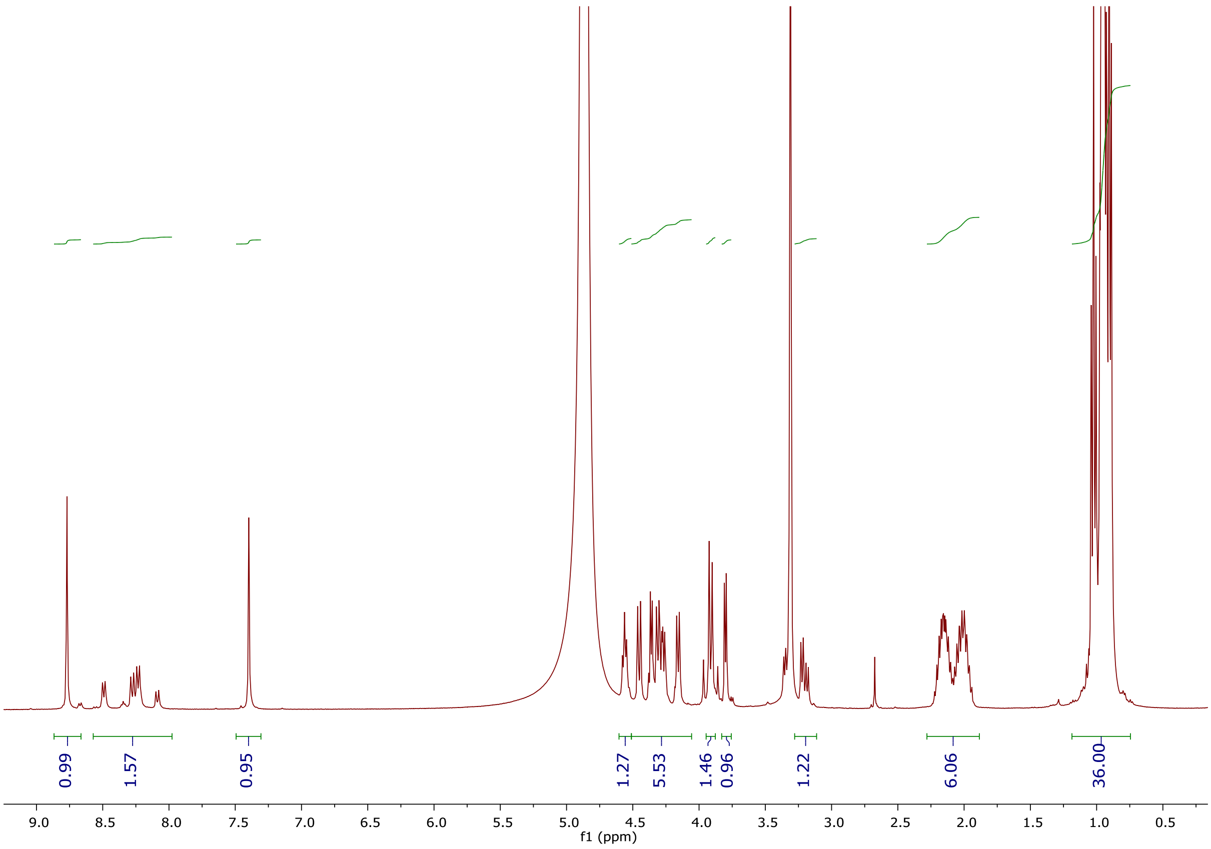

S 5 ^{1}H NMR spectrum of VVVGHVVV in D_2O at 400 MHz

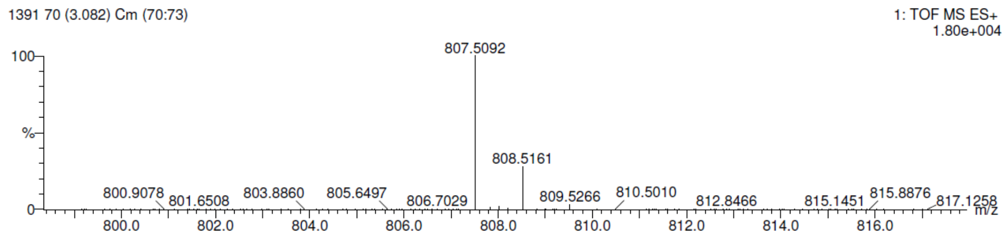

	Mass	Calc. Mass	mDa	PPM	DBE	i-FIT	Formula
Minimum:					-1.5		
Maximum:			5.0	3.0	20.0		
	807.5092	807.5092	0.0	0.0	10.5	1821.6	C38 H67 N10 O9

S 6 ESI HRMS of VVVGHVVV ion $[M+H]^+$

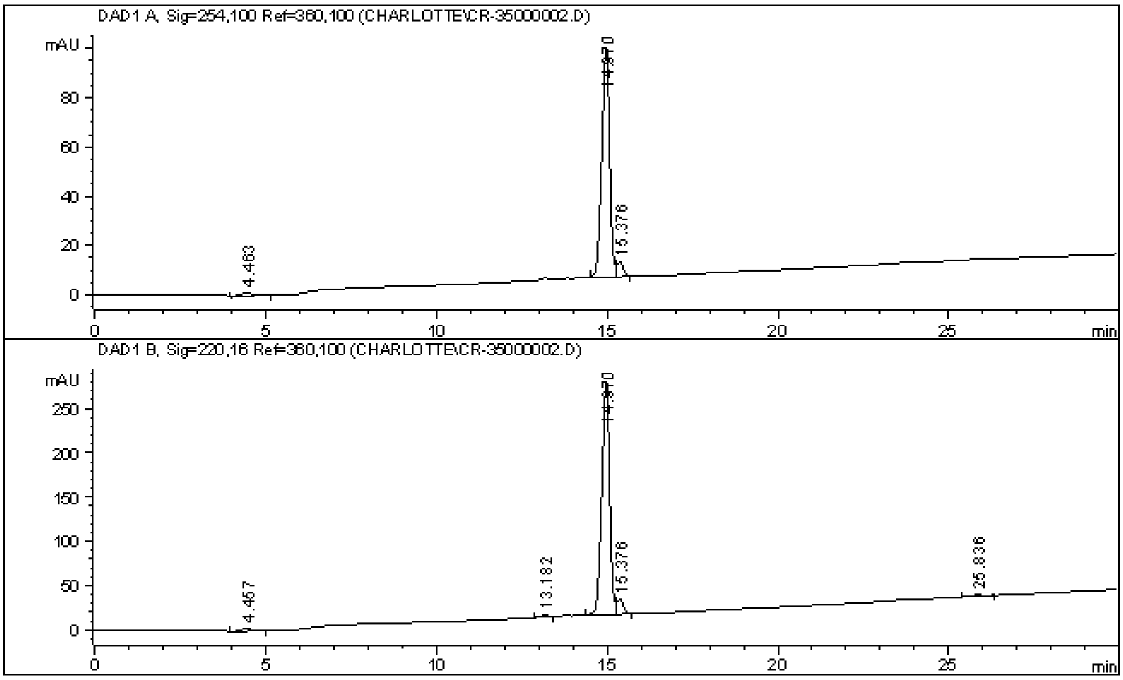

S 7 Analytical HPLC trace of VVVGHVVV. Gradient; MeCN (0.1% TFA): H_2O (0.1% TFA), 5:95 to 50:50 over 25 mins, 0.5 mL min^{-1})

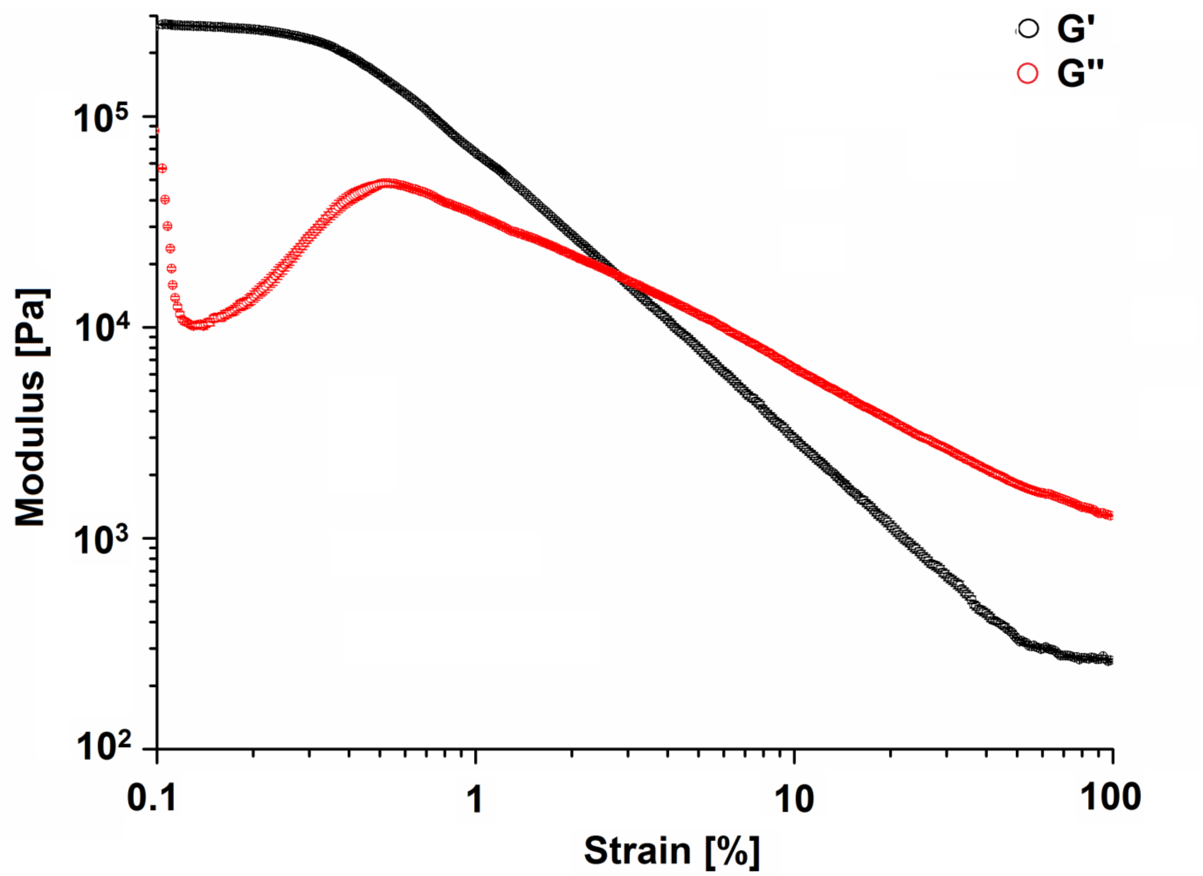

S 8 Storage (G') and loss (G'') modulus of a 0.20 wt.% (2.14 mM) solution of VVVGHVVV-C_8 in 50% acetonitrile/water as a function of strain. Error bars where visible represent two times the standard deviation from the log-averaged mean.

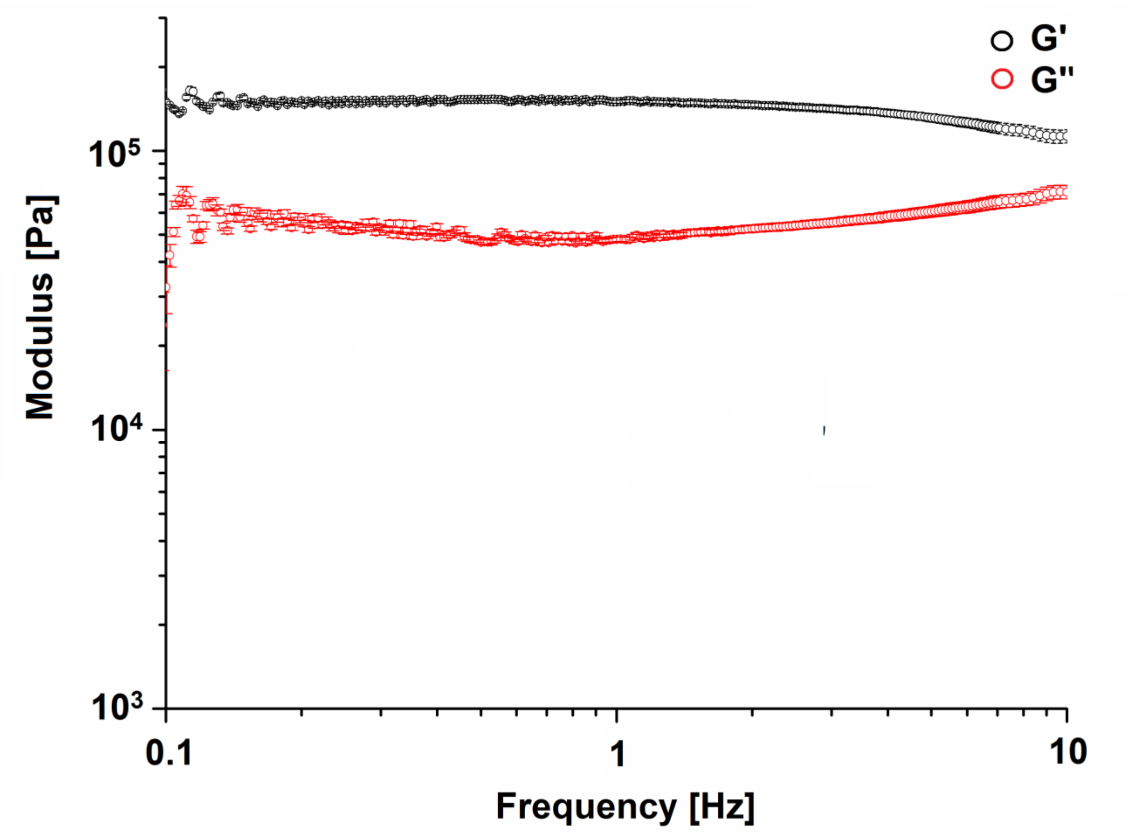

S 9 Storage (G') and loss (G'') modulus of a 0.20 wt.% (2.14 mM) solution of VVVGHVVV-C_8 in 50% acetonitrile/water as a function of frequency. Error bars where visible represent two times the standard deviation from the log-averaged mean.

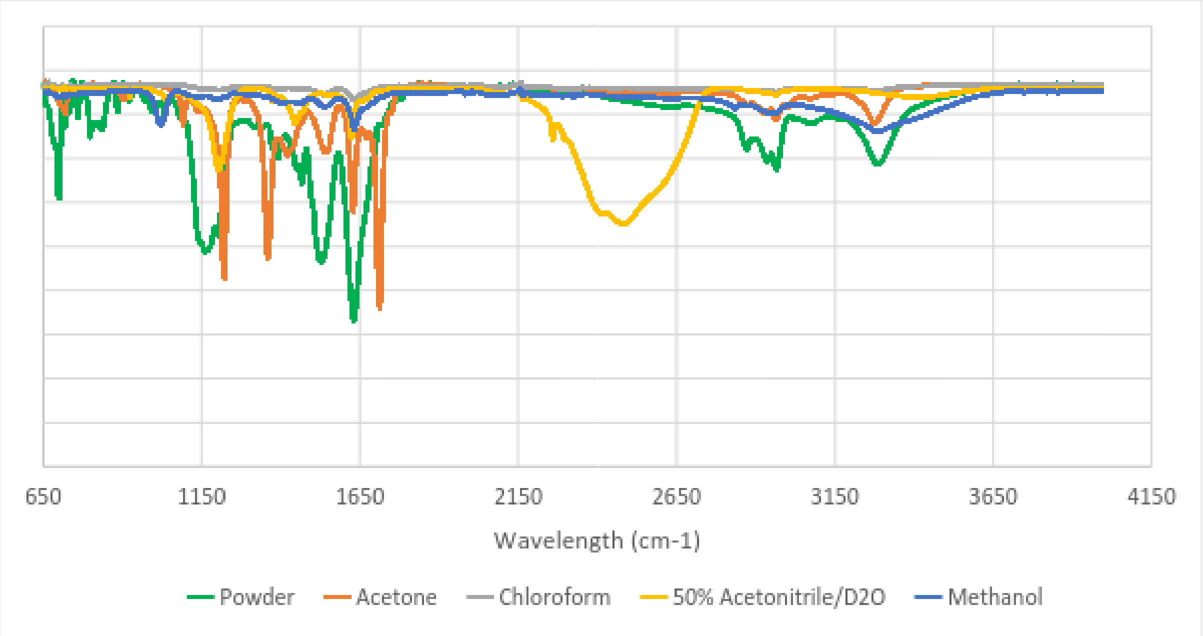

S 10 FT-IR spectra of VVVGHVVV-C_8 0.20 wt.% (2.14 mM) in acetone, chloroform, 50% acetonitrile/D_2O and methanol, and as a powder

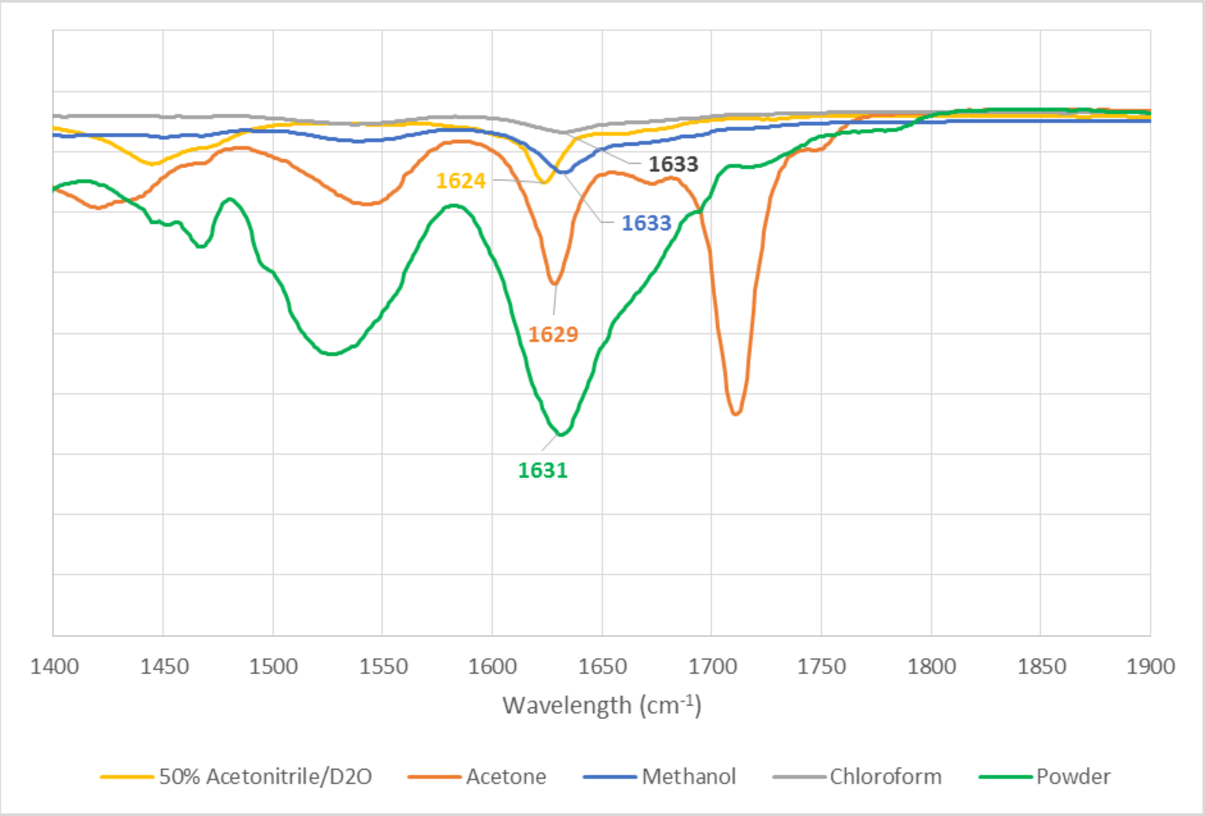

S 11 ®-Sheet carbonyl region of the FT-IR spectra of VVVGHVVV-C_8 0.20 wt.% (2.14 mM) in acetone, chloroform, 50% acetonitrile/D_2O and methanol, and as a powder

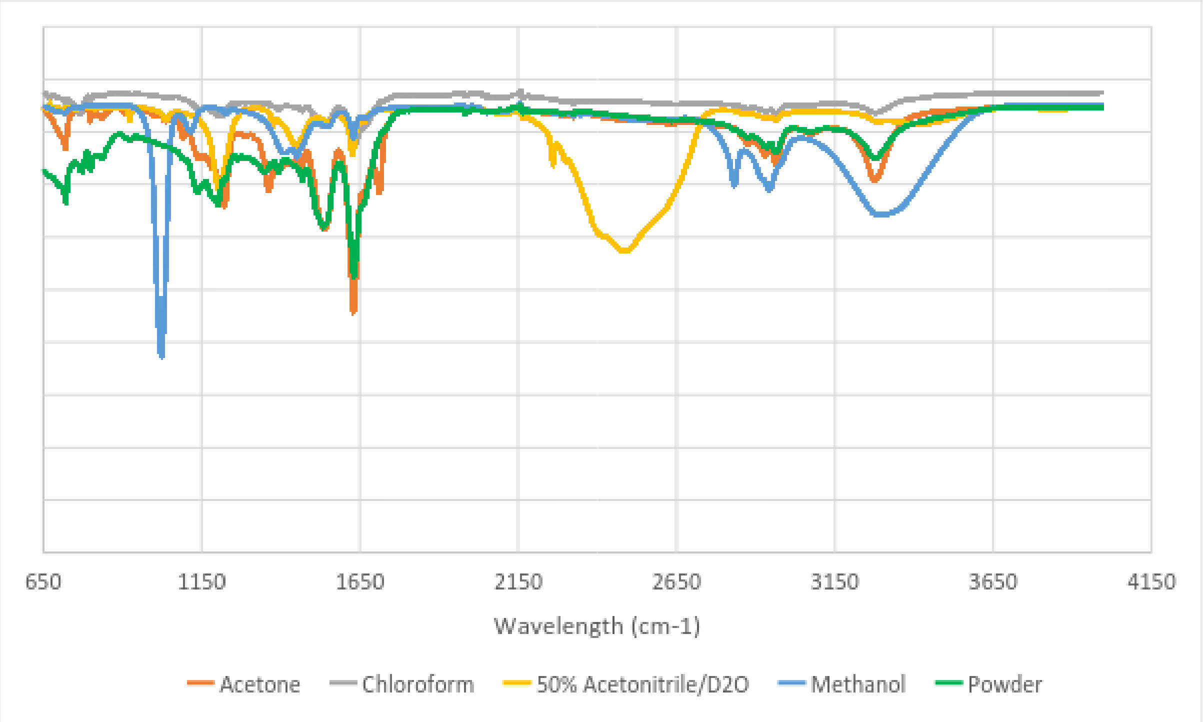

S 12 FT-IR spectra of VVVGHVVV 0.20 wt.% (2.14 mM) in acetone, chloroform, 50% acetonitrile/D_2O and methanol, and as a powder

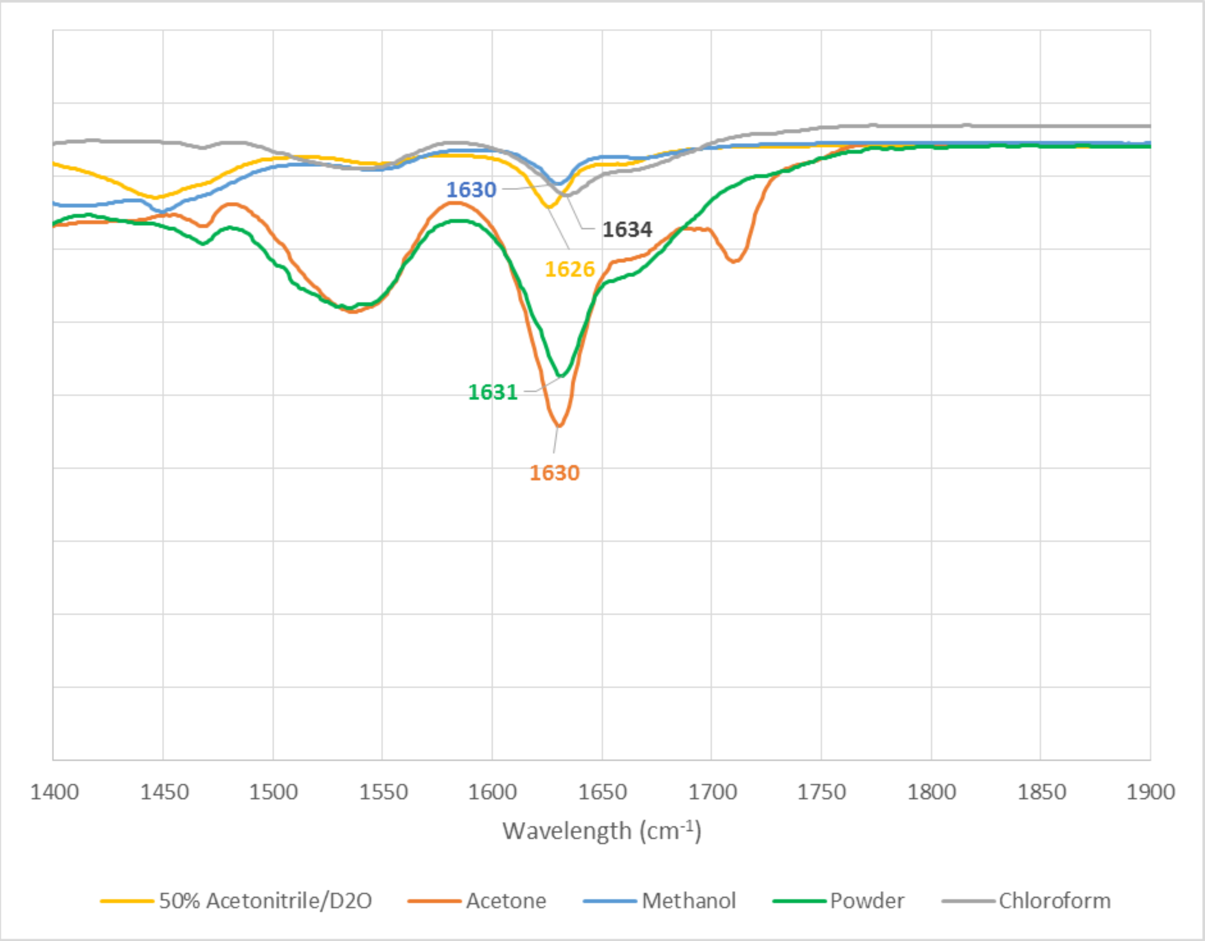

S 13 ®-Sheet carbonyl region of the FT-IR spectra of VVVGHVVV 0.20 wt.% (2.14 mM) in acetone, chloroform, 50% acetonitrile/D_2O and methanol, and as a powder

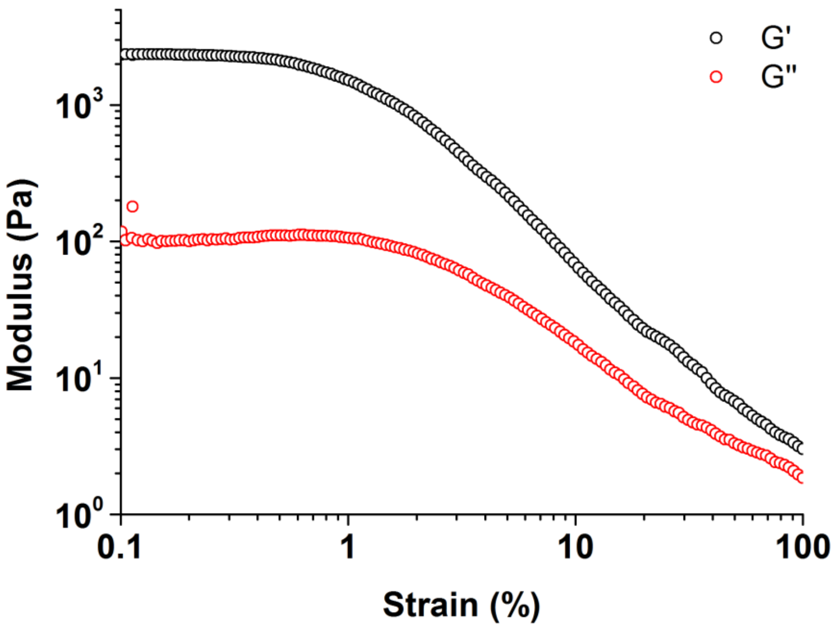

S 14 Storage (G’) and loss (G’’) modulus of a 0.20 wt.% (2.14 mM) solution of VVVGHVVV-C_8 in *N,N*-dimethylformamide as a function of strain. Error bars where visible represent two times the standard deviation from the log-averaged mean.

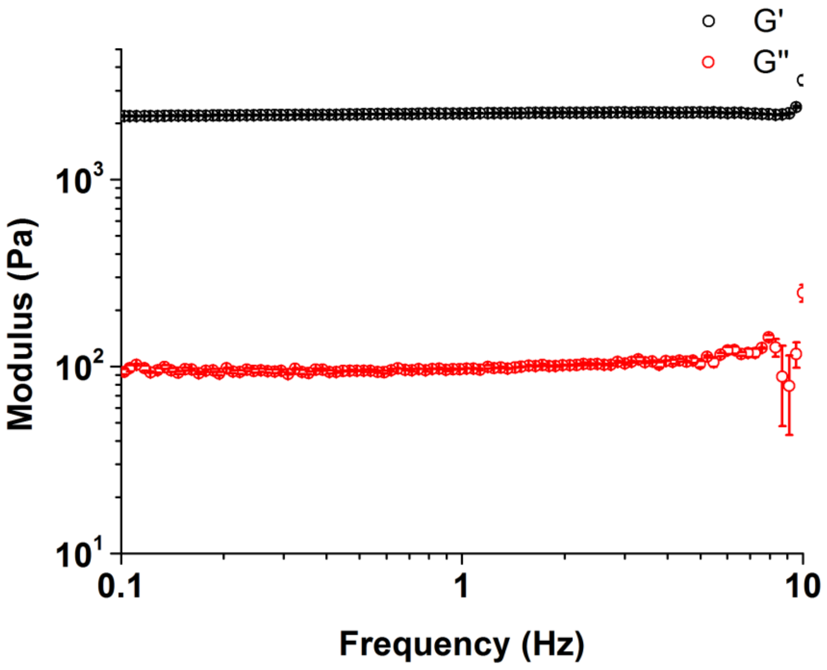

S 15 Storage (G’) and loss (G’’) modulus of a 0.20 wt.% (2.14 mM) solution of VVVGHVVV-C_8 in *N,N*-dimethylformamide as a function of frequency. Error bars where visible represent two times the standard deviation from the log-averaged mean.
